# Paid family leave on local television news in the United States: Setting the agenda for policy reform

**DOI:** 10.1016/j.ssmph.2021.100821

**Published:** 2021-05-15

**Authors:** Margaret Tait, Colleen Bogucki, Laura Baum, Erika Franklin Fowler, Jeff Niederdeppe, Sarah Gollust

**Affiliations:** a Department of Health Policy and Management, University of Minnesota School of Public Health, D305 Mayo Building, MMC 729 420 Delaware St. SE, Minneapolis, MN 55455 USA; b Wesleyan Media Project Wesleyan University, 45 Wyllys Ave. Middletown, CT 06459, USA; c Department of Government Wesleyan University, 45 Wyllys Ave. Middletown, CT 06459 USA; d Department of Communication Cornell University, 476 Mann Library Building Ithaca, NY 14853 USA

**Keywords:** Paid family leave, Local television news, Health equity

## Abstract

Access to paid family and medical leave (“paid leave”) has bipartisan support among lawmakers in the United States, but the issue remains stalled on the public policy agenda. The U.S. does not currently have a federal paid leave policy, and unpaid leave—guaranteed by the Family and Medical Leave Act of 1993—is all that is available to the majority of workers. In this study, we examine the content of local television news as representations of, and potential influence on, paid leave policy agendas. To do so, we analyze the extent to which local television news coverage describes the problem of lack of employment leave, and whether coverage highlights public policy as a solution. We use data from local television stations affiliated with the four major networks (ABC, NBC, CBS, and FOX) in all 210 media markets in the U.S. during a period pre-pandemic, from October 2018 until July 2019. We find that 64% of local television news coverage related to paid leave discussed the issue in the context of public policy. Coverage more often cited early-stage policy actions such as a policy idea - reflected in 40% of stories discussing stages of public policymaking – or the introduction of a bill – detailed in 22% of these stories. This coverage aligns with actual policy activity at the state-level during the same time period. News coverage infrequently included elements that could shape public understanding of paid leave as a population health issue, such as including health-related sources of providers or researchers. Policymakers, advocates, and researchers looking to advance public support for paid leave should consider efforts to use local television news as a vehicle to present health and policy-relevant information to broad segments of the public and set the agenda for policy reform.

## Highlights

• Local tv news coverage of paid family leave was infrequent.
• Relevant coverage highlighted policy details and included political sources.
• Political sources in coverage potentially politicized the issue for viewers.
• Details of the health equity implications of policy were largely absent.
• Local tv news outlets are important to prioritize in research dissemination.

## 1 Introduction

The U.S. is one of only a few developed countries without a national paid family and medical leave policy. Only 21% of U.S. employees working in the private sector, state government, or local government have access to paid leave through their employer (U.S. Bureau of Labor Statistics, 2020). Enacted in 1993, the Family and Medical Leave Act (FMLA) guarantees 12 weeks of leave and job protection upon an employee's return to work, but this leave is unpaid. When asked in January 2017 whether expanding access to paid leave should be a priority for the President-elect, 35% of U.S. adults responded that the issue should be a top priority, and 44% responded that the issue was important, though a lower priority than other issues (Pew Research Center, 2017). Candidates for office and elected officials have expressed increasing enthusiasm for expanding access to paid family and medical leave, and this holds across party lines—as demonstrated by recently introduced bipartisan legislation (Advancing Support for Working Families Act, 2019; New Parents Act of 2019, 2019). However, none of these federal policy actions have advanced beyond committee hearings.

While no federal legislation to expand paid leave has been passed since 1993 and the public has not perceived lack of access to paid leave as an urgent priority, the events of 2020 motivate public attention and policy action. The COVID-19 global pandemic offers a searing exemplar of the importance of social safety net policies that protect employees from financial hardship and allow them to care for themselves and their family (Ranji et al., 2020). The Families First Coronavirus Response Act included an Emergency Family and Medical Leave Expansion Act that offered eligible employees up to 10 weeks of compensated leave from their job (Families First Coronavirus Response Act, 2020) and the American Rescue Plan extends the availability of these provisions (American Rescue Plan Act of 2021, 2021). While this activity may represent critical first steps toward federal paid leave policy, legislation is temporary. In the absence of substantive protections for families through meaningful paid leave policy, it is important to understand the factors that may influence the policy agenda around paid leave and related policies.

One important influence on the policymaking process is the news media (Baumgartner & Jones, 2009; Kingdon, 1984). Local television news coverage is one important way the public and policymakers alike learn about social issues and the importance of policy action, as local television news continues to reach a larger and bipartisan audience than other print and national broadcast news outlets (Gollust et al., 2019). This study examines how local TV news media presented the issue of paid leave to the public in 2018–2019, a time period before the COVID-19 pandemic but still directly relevant to ongoing debates about leave policies that may emerge after temporary legislation expires. Below, we provide policy context around paid leave, describe how the news media can contribute to policy, and then discuss the study's objectives.

## 2 Background

### 2.1 What is paid leave policy?

**Fig. 1:**
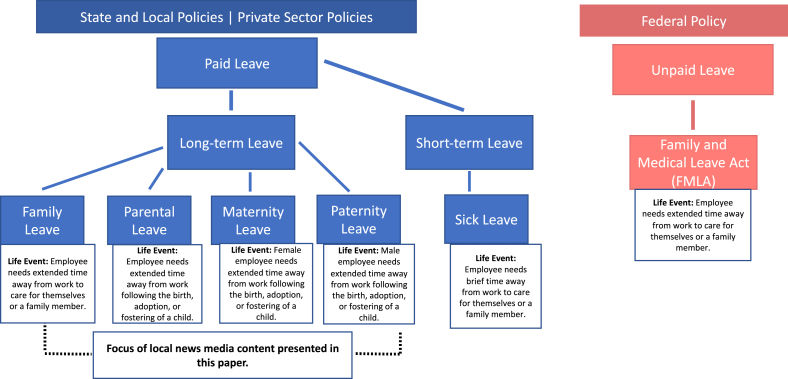
Typology of leave policies by level of enforcement.

While the components that encompass paid leave policy are broad, all have the same goal: to provide employees with compensated time away from work and job protection for taking this time off. Policies differ in the eligible life events for which an employee may take paid leave, which manifest in different policy nomenclature (e.g., paid parental leave, paid maternity leave). Fig. 1 visualizes a typology of leave policies by level of enforcement and the life events covered. At the time of this writing, ten states have passed a paid family leave policy, though not all have been implemented. A number of U.S.-based companies also have a paid leave policy in place for eligible employees. State and private sector policies share a goal of meeting a need where federal policy falls short, but state policies vary in how programs are financed, the amount of wage replacement they provide, length of leave, and conditions for benefit eligibility. For the purposes of this study, we focus on the broad category of paid family and medical leave, which we will refer to henceforth as paid leave.

### 2.2 Health and social impacts of paid leave

One rationale for enacting more robust paid leave policy comes from evidence of the pathways through which the use of paid leave can improve health for both individuals and populations. A limited number of U.S.-based studies have explored the health outcomes associated with paid leave policy, broadly. Existing studies and syntheses tend to focus on maternal and child health measures (Van Niel et al., 2020) or the evidence around the health benefits of parental leave (Gault et al., 2014). Impacts of paid family leave in this context include increases in birthweight, reductions in infant mortality, higher rates of breastfeeding (Mirkovic et al., 2016), and lower odds of re-hospitalizations for both mother and baby (Jou et al., 2018). For women, access to paid leave is positively associated with workforce participation; they are more likely to remain at a job after taking paid leave (Clark & Gallagher, 2017) and longer leave duration promotes better maternal mental health (Mandal, 2018). Evidence also reveals that access to and use of paid family leave is associated with better overall mental health for all those providing care to family members (Earle & Heymann, 2011; Rossin-Slater & Uniat, 2019).

### 2.3 Media coverage as a way of understanding the agenda for public policy change

The news media, through its established roles in society of informing, interpreting, and contextualizing content, remains an important way the public and policymakers learn about policy issues and proposed solutions (Graber & Dunaway, 2015). For instance, television news have an agenda-setting function; by reporting on some issues and not others, TV news signals to both the public and to policymakers about what are the most important issues of the day (Iyengar & Kinder, 1987; McCombs & Shaw, 1972). The media agenda can also contribute to the public agenda by emphasizing “the salience of issues, political figures and other objects of attention,” (McCombs et al., 2014). Understood in the context of paid leave policy, media coverage focusing on unpaid time away from work (the problem) or the benefits that come from having paid leave from work (a solution), could influence the public's and policymakers' opinions about the seriousness of the issue as well as the promise of solutions, particularly public policy solutions. Furthermore, the news media's reporting on federal or state policy action can provide information to the public that can help them advocate for change by providing them with context about legislation introduced and the types of stakeholders involved in advancing policy.

Local television news is a particularly important contributor to policy agenda-setting for a few reasons. First, more Americans trust local news than the national news (Gallup, 2019). For example, a 2020 poll found that 70% of Americans perceived local news as an important source in providing information during a crisis (Gallup, 2020). The public also prefers to access local news through television, as opposed to online, print, or radio (Pew Research Center et al., 2019). Second, for policy issues that are localized in nature (e.g., state paid leave policy action or municipalities’ leave ordinances), local news is likely to offer locality-specific information to audiences.

Despite the importance of local TV news, there are very few studies that explore its content. This is in part due to issues of access and resources: there is no comprehensive, public database of local TV news content (unlike those available for other types of print or national network TV news) and data must be purchased at a cost. A small number of researchers have examined the health-related content of local TV news (see Gollust et al., 2019 for a review) and suggest that local TV news does not provide a simple reflection of societal conditions. Instead, news organizations have their own influences (including advertising revenue), biases, constraints, and norms that contribute to programming decisions, including covering issues in ways that may sensationalize or entertain, instead of providing information about policy detail (Gollust et al., 2019).

### 2.4 Past research on news media coverage and paid leave

Limited scholarly work has focused on news media coverage of paid leave policy, and no study to date has examined paid leave policy in a nationally representative sample of local television news coverage. One research team has studied how the news media framed Senate Bill 1661 in California – which provided the groundwork for the first state-level paid family leave policy in the U.S. (Dorfman & Lingas, 2003). This study examined policy-related messages that appeared in local and national print media, select local television news outlets, national television evening news broadcasts, and a number of cable news programs. Although the study focused more on print media coverage, the authors note a key distinction between the individuals depicted as potential policy beneficiaries in print news as compared to television: “While the print news featured many stories about adults struggling to care for parents or other adult family members in addition to parents and children, the television coverage almost exclusively featured moms with newborns,” (P.20).

In the only peer-reviewed publication exploring news media coverage of paid leave, another research team analyzed print media coverage of Canada's Maternity Leave Benefit (MLB) (Dykeman & Williams, 2014). With insights from Kingdon (1984) on how policy issues garner attention on government agendas, the team analyzed media coverage as a way of identifying “what types of (policy) channels and pressures generated the opening of policy windows and the consequent evolution of the MLB.” (P.6). This research underscores motivation for the current study: that insights from media coverage of different types of leave policy can provide ideas of how related policy can be presented in the media to garner the public's and policymaker's attention.

The current manuscript seeks to fill a large gap in past research: to illuminate the characteristics of local TV news media coverage of paid leave policy. We implemented a content analysis of local television news coverage to quantify the prevalence of paid leave in news coverage and describe the policy context. We prioritized coding variables that provide evidence of the type of leave referenced in coverage; the use of personal stories to exemplify who could or should benefit from paid leave; the extent to which coverage was politicized or included policy-relevant information; if policy-relevant, where a policy was in the policymaking process; and (again if policy-relevant) whether or not coverage described local policy activity. Findings contribute to growing scholarship on the role of local TV news in informing public understanding of health issues, particularly the social determinants of health. The findings also reveal current and future opportunities for both advocates and policymakers to pursue equitable paid leave policy.

## 3 Materials and methods

### 3.1 Data and sampling

Data for this study were purchased from TVEyes, a commercial search engine for TV and radio coverage. Our first task was to develop a set of keywords to search closed captioning of local TV news broadcasts in order to capture a sampling frame of stories concerning paid leave policy. Recognizing the broad universe of paid leave policy, the research team developed a set of terms to employ as keyword search terms (see supplementary appendix 1). Searches were implemented on closed captioning transcripts of broadcasts that aired from July 30, 2018 to July 31, 2019 and yielded 22,929 total hits of news content with one or more keyword. While we had keyword hits for the full time period, due to resource constraints we only downloaded video files for stories that aired from October 1, 2018 onward; therefore, we drew a sample designed to be representative of local TV news that aired between October 1, 2018 and July 31, 2019.

### 3.2 Sampling construction

As a second step, we used a constructed week sampling approach to arrive at a manageably sized, probability-based sample. Since we wanted to examine the full videos (and not just transcripts) for imagery used in stories, we used a constructed week sampling strategy for October through July, the period with available downloaded video files. Constructed week sampling is a validated stratified random sampling technique wherein the final sample represents all days of the week in effort to account for cyclic variation of news content (Luke et al., 2011). For this study, our interest was in analyzing news coverage that aired on broadcasts with both high viewership (Pew Research Center et al., 2019) and when the research team hypothesized there may be more substantive content. Thus the constructed week did not include weekend days and only included hits from early evening (4–7p) and late evening (8–11:59p) broadcasts, thereby excluding local morning TV news shows which tend to feature weather and traffic reports to a greater extent than evening broadcasts and as a result, may be less likely to include policy-relevant content (Jurkowitz et al., 2013). To further refine the sample, we excluded hits from local news stations not affiliated with the four major networks (e.g. ABC, NBC, CBS, and FOX), which are only available in select markets. Our constructed week sample included 218 days of content comprising 320,460 broadcasts and yielded a sample of 3024 hits ahead of the content analysis stage.

To refine the sample for content analysis and narrow to stories related to research objectives, the third step, coders were directed to exclude keyword hits that appeared in video stories that were not relevant: advertisements that aired during the commercial breaks of local news, teasers that signaled upcoming content in a local news broadcast, and hits for “childcare”, which were included in the sampling frame for a separate content analysis focused on early care and education. Once eliminating those stories, there were 1275 stories that were analyzed for content. Additional exclusions were applied during the content analysis stage to yield the final analytic sample of stories focusing on or mentioning paid leave (see Section 3.4).

### 3.3 Content coding and content instrument development

Considering available information about public and private policy discussion and politics around paid family leave, the research team developed a content analysis instrument to capture details of local TV news coverage. To begin, coders identified whether the story included substantive discussion of paid leave (signaling what we refer to as a focus-level story) or simply a passing reference (termed a mention-level story). We then coded all stories for the following core variables: type of leave referenced; an indicator of whether policy was discussed and if so, details about the policymaking process; and the locations mentioned relevant to paid leave. We also coded focus-level stories for political actors and other sources heard speaking or visualized in coverage, and whether there were exemplars included.

Coders then identified stories that included an explicit policy orientation, discussion of paid leave in the context of local, state, or federal public policy or budget processes. Discussion of paid leave in the context of public policy signals to the public that the issue is one for government to consider and not solely a problem of the private sector or family concern. References to paid leave in the context of an executive branch (e.g., Governor's) budget also signals that the issue is within the realm of government responsibility. Stories that included a reference to public policy were subsequently coded for the stage of policy making process, if any, that was referenced to provide some idea of whether local news was covering the paid leave policy early in development, in later stages, or throughout the process. In parallel with the content coding, we also searched legislative databases to identify state-level policy activity during the 2018–2019 study period in an effort to consider how attention to paid leave in news coverage relates to actual policy activity. In addition to coding for the stage of the policy making that might have been discussed, coders were also asked to select if policy context was provided, such as detail about the amount of benefit that would be provided through a policy and who would be eligible, or how the benefits would be financed.

Next coders identified any political sources and their partisan affiliation if referenced, as well as other individuals included as sources in the context of paid leave and their positions on leave. Understanding that partisan and other source cues impact public and policymakers' attention to issues (Druckman et al., 2013) and may shape their support for policy change, we attempted to quantify and describe the cues used in the context of paid leave to better understand which stakeholders’ voices were or were not incorporated in coverage.

Another policy-relevant variable is whether the news stories included exemplars or stories about identifiable individuals, as such depictions can influence policy-relevant attitudes among the public (Barry et al., 2013). Consistent with scholarship on exemplification theory (Zillmann et al., 1996), we defined an exemplar as a person identified on screen or orally by a reporter or other speaker, and the speaker or the person themselves referenced their experience with paid leave (either benefiting from or lacking). A team of trained coders conducted a secondary analysis on all stories (N = 28) identified as visualizing an exemplar and visually inspected the gender, role, and race of each exemplar. Of this sample, three were excluded because the policy discussed in coverage was sick leave and not the broader leave policy relevant to our analyses.

The research team was also interested in understanding if local news coverage of paid leave was local in scope. Stories that included a location reference received additional coding to identify the specific location referenced. Secondary analysis was performed to compare the location mentioned to the verified catchment area of the media market in which the clip aired.

Coders performed this content analysis on the final sample of stories that were identified from the keyword hits (n = 557) as mentioning or focusing on paid leave. A subset of stories in the entire sample (N = 425, 33% before applying exclusions) were viewed by multiple coders and the level of inter-coder reliability agreement was assessed by calculating Krippendorf's Alpha. As the variables included in this study were binary and ordinal, Krippendorf's alpha is a more flexible measure of inter-coder reliability than other metrics (Hallgren, 2012). The analysis presents only those variables with an alpha above a minimally acceptable threshold (0.65). The variables presented in this paper have alphas ranging from 0.65 to 1, with an average alpha of 0.83. A list of the ICR values for each variable is included in supplementary appendix 2.

### 3.4 Final analytic sample exclusions

Supplementary appendix 1 is a flow chart visualizing the process for arriving at the final analytic sample comprising stories focusing on or mentioning paid leave. We excluded stories captured in our sampling approach (see 3.2) but that did not include any mention or focus on paid leave (N = 75) or that mentioned or focused on an individual taking leave without a connection to broader discussion of policy (N = 300). We implemented additional exclusions for stories focusing only on animals (e.g. references to “pawternity” leave) as well as stories wherein the keyword hit was in relation to the 2019 government shutdown. The latter exclusion was motivated by our observation that coverage of the shutdown was episodic in nature, discussing the issue of leave only in the context of federal workers and during a specific time period, and not relevant to the broader population health issue of paid leave. Overall, 306 stories mentioned paid leave in passing as part of a larger story, while 251 stories focused on paid leave and included substantive coverage, for a total of 557 stories in the final analytic sample. Considering the potential for one story per broadcast to include a story focusing on or mentioning paid leave, this number reflects a very small proportion of the possible broadcasts (n = 320,460) in the constructed week sample that could have included a story meeting our criteria of sufficient relevant content to have been included in this study.

## 4 Results

### 4.1 Summary of sampling frame – total news hits

Supplementary appendix 3 depicts the volume of keyword hits in all 210 media markets for the data collection period we had video files for, October 1, 2018 to July 31, 2019. These hits include stories that were ultimately excluded but are included here to provide an idea of overall volume.

**Fig. 2:**
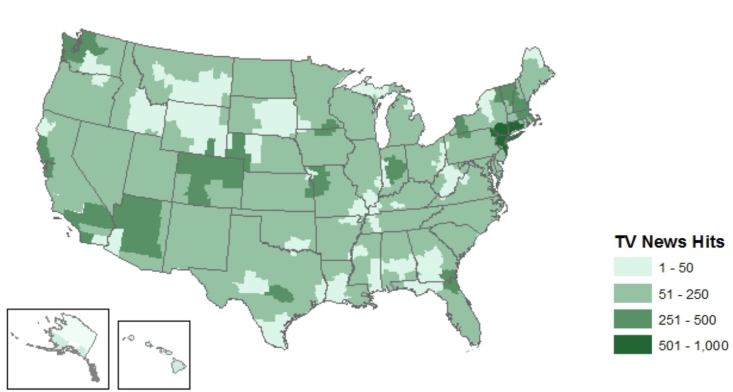
Volume of paid leave keyword hits from 07/30/18 to 7/31/19 by geography.

Fig. 2 is a map of total keyword hits during the keyword hit data collection period, July 30, 2018 to July 31, 2019 in each media market. Of note, this visual reflects all of the keyword hits, including those excluded from the analytic sample, so this is an upper bound of possible coverage. The figure reveals the highest volume of attention to paid leave in media markets located in the northeast, Washington, California, Arizona, and Colorado.

**Fig. 3:**
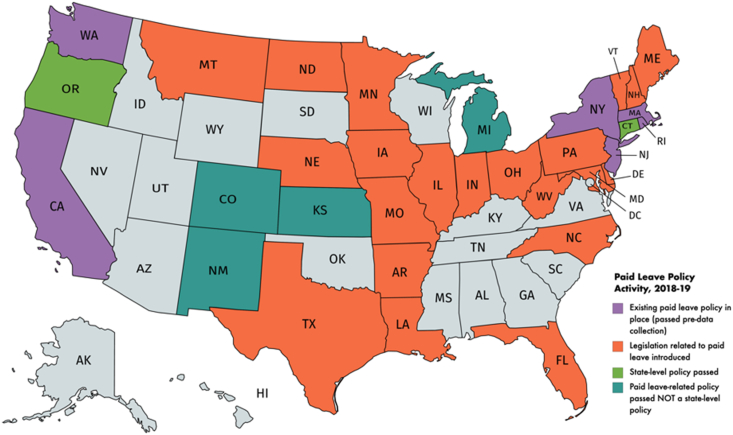
State-level paid leave policy activity, 2018-19.

Fig. 3 depicts state and, to some extent, local-level paid leave policy activity during legislative sessions in 2018 and 2019 that coincide with data collection. At the start of data collection, 6 states and the District of Columbia had passed state-level paid leave policy; of the 44 states without a policy in place, 24 had related state-level activity during data collection and 4 states passed paid leave policy relevant to a segment of the population or a specific type of leave. These four states are indicated in blue, within the category of “Paid leave-related policy passed NOT a state-level policy”. An example of the latter category would be Kansas Governor Jeff Coyler's executive order to provide paid parental leave for Kansas state employees (Kansas Department of Administration, 2018). This is likely a conservative estimate of more localized activity as the data only reflect state-level legislation to explore or implement policy and do not include municipalities activities (e.g. efforts in Austin, Texas to pass paid sick leave).

### 4.2 Extent and type of leave referenced in local TV news coverage

Table 1 summarizes the type of paid leave explicitly referenced in all stories in the sample for content analysis. Stories could have included references to more than one type of leave and for analysis, our universe of stories referring to paid leave includes all stories except those referring only to sick leave. Stories more often explicitly referred to family leave (n = 258). Fewer stories in the analytic sample explicitly referred to paid leave (n = 107) and a large number referred to sick leave (n = 193). Stories referring only to sick leave were excluded from further coding because the research interest was in policies providing a longer duration of leave. Keyword hits resulting from discussion of an individual's leave experience (n = 300) such as a reporter returning to work after paternity leave were excluded based on our observation that these stories infrequently discussed paid leave, broadly.

**Table 1:** | Type of leave explicitly referenced in local news stories.

	N (%)
	Total = 557
**Paid leave**	107 (19.2)
**Family leave**	258 (46.3)
**Medical leave**	127 (22.8)
**Sick leave**^b^	193 (34.7)
**Parental leave**	83 (14.9)
**Paternity leave**	9 (1.6)
**Maternity leave**	41 (7.4)

b Stories mentioning or focusing only on sick leave were excluded from further content analysis.

Table 2 presents the prevalence of references to paid leave as part of a local, state, or federal public policy or budget in coverage. Policy was a major element of coverage: nearly 64% of stories discussed paid leave in the context of public policy.^1^ Far fewer (3.6%) referenced paid leave as part of a budget. Stories that included a reference to paid leave in the context of public policy (n = 355) received additional coding intended to identify if details of the policymaking process were included and if so, the stage of the process discussed. Of the stories discussing paid leave policy, almost half (45.9%) included details about the policymaking process (Table 2).

**Table 2:** | Policy-relevant content in news stories about paid leave.

	N (%) Total = 557
Stories referencing public policy	355 (63.7)
Stories referencing a governmental budget	20 (3.6)
Details about process included^b^	163 (45.9)
**Stage of the Policy Process**	
**Total** = **163**	
Policy idea	55 (33.7)
Draft bill introduced, discussed	101 (61.9)
Passed a vote	3 (1.8)
Did not pass a vote	0 (0)
Going for executive approval	0 (0)
Signed into law	8 (4.9)

b Code was asked of stories referencing policy (N = 355).

The majority of stories that provide details of the policy making process referenced early stages of the policy making process; paid leave was referred to as a policy idea in 33.7% and as draft bill that was introduced in 61.9% of these stories. Here, too, more than one code could be selected. None of the stories discussing policy signed into law (n = 8, 4.9%) detailed activity in Oregon or Connecticut, the two states that passed state-level policy during data collection. This variable captured any paid leave policy that was signed into law, and stories in our sample reflect more localized policy activity (e.g. policy providing leave to one type of worker, such as firefighters). Related to policy context provided in stories, a small number of focus-level stories (n = 20, 7.9%) included details about how policy would be financed through new or existing taxes. Slightly fewer (n = 12, 4.8%) referenced specific employees who would be eligible for benefits. Our codebook included variables intended to capture additional characteristics of policy beneficiaries explicitly discussed in coverage, such as whether affected individuals were described in terms of geography, race, income, or sexual orientation, but these were infrequently observed and the inter-rater reliability was low.

### 4.3 Sources

Table 3 presents the partisan sources heard speaking and/or visualized in stories that focused on paid leave and the positions these sources expressed. Close to half (46.2%) of stories focusing on paid leave included a political source, an individual identified visually or audibly with their party affiliation. The only politician coded as a political source absent visual or audible identification was President Trump, since we assumed that most viewers would know his party affiliation without explicit reference to it. President Trump was featured expressing support in about fifteen percent of stories and a mix of support and opposition in a smaller percentage (4.7%). Stories focusing on paid leave more commonly included a Democratic lawmaker (31.8%) and Democrats more often signaled their support in stories (18.7%) than a mix of support and opposition (13.1%). More than a third of stories focusing on paid leave featured a Republican or Trump (35.8%) and more of these stories included Republicans or Trump signaling their support (21.5%). We identified no stories that featured any partisan group expressing exclusively opposition to paid leave.

**Table 3:** | Partisan sources included in news stories focusing on paid leave.

Partisan Source (% in stories) N = 251	Support (%)	Opposition (%)	Mix of support/opposition (%)
Any of Trump, Republican, or Democrat (46.2)	80 (31.8)	0	36 (14.3)
President Trump (19.1)	36 (14.3)	0	12 (4.7)
Democrats (31.8)	47 (18.7)	0	33 (13.1)
Republicans (24.3)	28 (11.2)	0	33 (13.1)
Republicans or President Trump (35.8)	54 (21.5)	0	36 (14.3)

Coders were also prompted to identify the positions (in support, opposed to, or a mix of support and opposition) of other sources expressed related to paid leave (Table 4). We used the term “elected official” as a catchall for any individual elected to public office. Unlike the earlier codes used to identify the audible or visual presence of a politician specific to their party, and subsequently, partisan cues, these data do not break down by party because a partisan cue was not likely to be presented in coverage, and as so, signaled to a viewer. The majority of elected officials included in stories who indicated a position on paid leave voiced only support (40.6%), while less than two percent (1.6%) indicated only opposition. Doctors, nurses, or other professionals representing health and health care sectors, researchers, and social service providers were not featured in any stories within the sample. Related, while we initially set out to code for the frequency of use of health-related arguments used in support of paid leave policy in stories, such arguments were too infrequent and the resultant inter-rater reliability too low to capture this messaging.

**Table 4:** | Other sources included in news stories focusing on paid leave.

Source (% in stories) N = 251	Support	Opposition	Mix of support/opposition
Any elected official (58.2)	102 (40.6)	4 (1.6)	39 (15.5)
Government representative, non-elected official (15.5)	39 (15.5)	0	0
Advocate, lobbyist, interest group (6.4)	16 (6.4)	3 (1.2)	2 (1)
Regular person/community member (8.0)	20 (7.9)	0	2 (1)
Researcher (0)	0	0	0
Doctor, nurse, or other health care professional (0)	0	0	0
Social worker/social service provider (0)	0	0	0

### 4.4 Exemplars | individuals depicted as current or potential policy beneficiaries

Overall, 34 stories (13.5%) that focused on paid leave included an exemplar and signaled a potential policy beneficiary. Of these, 23 of the stories visualized an exemplar and discussed paid leave (not sick leave) and thus were included in a secondary analysis to examine the demographic characteristics of those visualized. A few stories included more than one exemplar. As so, we present select values as a percentage of stories with an exemplar and others as the percentage of all exemplars visualized in these stories; where applicable we note the percentage of a specific type of exemplar (e.g. mothers). The majority of stories with an exemplar (91.3% of stories) highlighted women who were mothers. Among mothers depicted in stories (90.6% of all exemplars) most were identified by coders as white (75.8% of mothers included as exemplars). People of color (21.8% of exemplars) and individuals identified as caregivers (3.1% of exemplars) or fathers (3.1% of exemplars) were less frequently a part of coverage. Incidence of exemplars in the sample were not always unique; many of the same stories appeared more than once in the sample in packaged segments.

### 4.5 Location represented in news coverage

The majority of all stories (78.1%) referenced a location in their discussion of paid leave, noting a city, county, region, state, or the U.S. or another country. Among stories that included a location reference, 22.8% noted a location outside the catchment area of the media market and 7.4% referred exclusively to such a location, like a story airing in the Topeka media market that focused on efforts to pass parental leave in North Carolina. Stories may have included references to more than one location and few stories (13.9%) included this type of comparison to other locations.

## 5 Discussion

This study is the first to our knowledge to examine a nationally representative sample of local TV news content related to paid leave in the U.S. Our results provide evidence that paid leave is infrequently discussed on local television news –our analytic sample included just 557 stories that mentioned or focused on paid leave across 320,460 broadcasts in our constructed week sample. Despite the low overall volume, coverage goes beyond focusing on the problem of access to paid leave and includes discussion of the policy solutions needed. A sizable portion (63.7%) of stories in our sample included a reference to public policy. Stories referencing public policy that also highlighted details of the policymaking process (45.9% of this subset of stories), more often referenced early stages of the process, such as a policy idea or a draft bill going for review. This tracks to legislative activity during our data collection period (Fig. 3): Paid leave was frequently part of state-level legislative activity, but policy was infrequently passed. In this way, local television news reflects paid leave policy activity.

It is worth emphasizing that paid leave policy remains confusing to many people (Lerner, 2010) and the uptake of benefits newly available as part of COVID-19 relief packages has been low (Miller & Tankersley, 2020). Local TV news coverage in our sample was very unlikely to present paid leave content that could aid in viewers’ understanding of important policy details related to who would be eligible for benefits and what these benefits would provide, as fewer than 1 in 10 stories offered such content. Our analytic sample comprised more stories that mentioned paid leave in a passing reference, such as a story detailing the priorities of a candidate running for office or providing a legislative update. Less than half of stories (45.1%) covered more substantive content related to paid leave, including details that could influence opinion on the necessity of policy reform. This information, provided in the pre-pandemic period, could have primed viewers to understand benefits available to them as result of the pandemic.

In the stories with more substantive coverage of paid leave, political entities were most often the individuals visualized or heard speaking and thus signaled to the public as an information source on the issue, albeit a partisan one. Considering the high percentage of stories with references to public policy, it is understandable that candidates, elected officials, or government officials would be included as part of coverage. Paid leave remains a topic that has bipartisan support (and our data demonstrated that no politicians signaled exclusive opposition to paid leave). Individuals representing the business community, advocacy or interest groups, and lay people were all less likely than politicians to appear as sources in coverage. This is surprising considering representatives from the business community have been publicly opposed to various paid leave policies (Noguchi, 2017).

We did not observe researchers, health professionals, or social workers represented in local news stories in our sample. Portrayal of these sources could offer an opportunity to shape viewers' understanding of the broad implications of the issue and solution, such as for population health, well-being, and equity. Scholars spanning different disciplines have conducted research on the prevalence and impacts of paid leave policy (see, e.g. Doran et al., 2020; Itum et al., 2019) and this evidence could play a role in shaping viewers’ understanding of the merits or potential downsides of policy reform—yet these perspectives are absent in local TV news.

Inequities in access to paid leave persist. Across all types of paid leave, more white people report access than non-white Hispanic people, and across all types of leave except for leave-taking related to child care, white people report greater access than Black people (Bartel et al., 2019). In an effort to understand different ways in which local news coverage included signal of disparities or the opportunity for policy to promote equity, coders attempted to code variables designed to capture discussion of how paid leave policy could benefit specific groups. These variables included racial and ethnic populations who have systemically been marginalized from access to public policy benefits (C. M. Grogan & Park, 2017; Grogan & Patashnik, 2003). We observed few stories in our sample that included details of groups that could benefit from policy and even fewer that explicitly discussed aspects of group identity such as race and others related to equity (e.g., sexual orientation and rurality).

Analyses of the exemplars included in coverage revealed limited inclusion of non-white and non-maternal figures. Considering exemplars as an implicit message about the target population for paid leave policy, such a narrow focus on parental leave and among white women is counter to messaging that advocacy groups suggest to build support for policy reform. Recent work reveals that individuals respond better to stories that emphasize the opportunity for paid leave policy to mitigate the burden on caregivers, as a broadly defined population, and not solely individuals with parental responsibilities (The Opportunity Agenda, 2019). Stories without details of populations that stand to benefit from paid leave and that neglect to highlight the diversity of individual stories limit the potential for news coverage to bolster political will in support of policy change or to further public understanding of all who need to benefit from policy reform.

Our content analysis offers lessons for scholars seeking to understand the opportunity for local television news to convey health information to the public. The first lesson relates to the cues included in coverage that could limit viewers' understanding of important health and social policy issues. Local news television coverage of paid leave in our sample more often included political entities than other types of sources. Coverage including this high volume of partisan cues may contribute to viewers’ interpretation of the issue of access to paid leave as politicized. Other research has demonstrated that partisan cues in news can reinforce polarized opinions about policy issues (such as the Affordable Care Act) and limit the extent to which new information can contribute to learning and attitude change (see, e.g.,Druckman et al., 2013; James & Van Ryzin, 2017; Gollust et al., 2020).

Our results also provide evidence supporting the conclusion that local television news outlets continue to struggle to cover issues related to the social determinants of health (see, e.g., Gollust et al., 2019). Paid leave – a social policy – impacts individual and population health (see Rossin-Slater & Uniat, 2019 for a summary of health impacts) and the opportunity for linkages between paid leave policy and health abound. However, we observed essentially no connections between paid leave and health or health-related sources interviewed in our sample of local TV news. There are many reasons for this dearth of coverage, but those relating to local television news resources and elements of news production are worth emphasizing. Journalists tasked with reporting on state and local policy issues may not be familiar with the social determinants of health or health equity, just as some health journalists might focus more on health care policy and not on the broader health determinants. TV news reporters and producers may find it difficult to apply a health equity lens in their reporting, given the time it would take to fully research the issue (beyond a brief policy update) and find appropriate sources to appear on screen (see, e.g.Marvel et al., 2018). Further, news norms and the demand to provide information in a compelling way in a very short format emphasizes novelty over persistent structural issues (Gollust et al., 2019).

Not least in importance, our analysis offers lessons relevant to those interested in the changing dynamics of local media outlet ownership. Efforts to consolidate local television news stations have captured national attention, recently with the unsuccessful attempt by Sinclair Broadcasting Group to acquire Tribune Media Group (Lee & Tsang, 2018) and ongoing attention to how local TV has become more nationalized and conservative-leaning with these ownership changes (Martin & McCrain, 2018). These attempts and other trends in local media outlet ownership raise questions of if and how changes in station ownership impact the content of local news (Hedding et al., 2019) as well as the scope of that coverage. We found that a subset (7.4%) of the stories analyzed for content in our sample discussed activity that was not actually local. A focus away from local stories toward more nationalized content curbs the potential for news to cultivate values, beliefs, or policy support applicable to local communities.

## 6 Limitations

Our analysis reflects only a small (albeit representative) subset of paid leave-related coverage during the study period, per use of constructed week sampling, and is limited by the categories we chose to capture in our content analysis. Further, because we applied a quantitative and finite content analysis instrument, our analyses do not represent the universe of coverage or other potentially important elements of paid leave news coverage, such as a more thematic or qualitative assessment of the messages or values used in building support (or opposition) to paid leave as a policy issue. Our content analyses instrument included only a selection of messages measured quantitatively (e.g. whether or not messages appeared such as that paid leave policy promotes health; paid leave policy is bad for the business community) and these messages were infrequently observed in our sample, suggesting that messages we prioritized may not have been used in local television news or that different messages were. Further, when coding for the race of individuals included as exemplars, we only included two categories: white or person of color. People of color are not monolithic and such a binary limits what we can infer about populations of color included in coverage and the extent that coverage provided signal of populations that could or should benefit from policy reform. That said, coverage of non-white exemplars was very rare in the sample to begin with, and it is very difficult for a coder to judge a person's racial identity in nuanced terms. Taken together, these constraints limit the extent to which the current study can inform discussions about specific arguments that are employed in public discourse related to paid leave policy, and to what extent paid leave policy is discussed in terms of health (in)equity. However, we are sufficiently confident in the scarcity of news coverage that employed health equity arguments or references to groups that could benefit from policy action that we can conclude health equity was not a frame local news reporters used in their coverage.

## 7 Conclusion

Just 6 months following the conclusion of our data collection in July of 2019, the U.S. was thrust into the COVID-19 global pandemic. During this time, more adults in the U.S. report paying attention to local news than in previous years (Gallup, 2020). The virus presents an immediate need for employees to have paid time off from work to recover from the virus or to care for loved ones. As such, paid leave is significantly more of a priority than it was previously and the need for public support and political will to achieve reform is greater. The policy response to the COVID-19 global pandemic has included provisions related to paid leave, but protections are temporary and have been implemented in such a fragmented way that many who could benefit remain confused and uptake has been low (Miller & Tankersley, 2020).

Our analyses reveal that a large proportion of local news coverage of paid leave pre-COVID-19 referenced public policy and could be taken as a positive signal of local television news’ capacity to set the agenda for policy reform beyond temporary measures. Our analysis suggests there is opportunity for local news media to provide more details about paid leave policy—focusing longer-form stories on paid leave, including details about policy that could bolster public support, elevating the experiences or needs of people diverse in race, ethnicity, and occupation, and highlighting policy activity that is local in scope. These details were infrequently observed in our sample, which included a small number of stories about paid leave in comparison to the amount of content in our sample that could have been about paid leave. Health researchers and advocates alike have an opportunity to forge better connections with local TV journalists in order to achieve more attention to paid leave as a population health-relevant issue. It remains to be seen whether future periods of news coverage can address these missed opportunities to communicate about policy details and potential beneficiaries, as disparities in access to paid leave persist in the wake of the pandemic.

## Author statement

Margaret Tait – conceptualization; Writing – original draft; Formal analysis Colleen Bogucki – conceptualization; Writing – review & editing; Supervision; Project administration; Data curation Laura Baum – conceptualization; Data curation; Visualization; Writing – review & editing; Funding acquisition Erika Franklin Fowler – conceptualization; Writing – review & editing; Resources; Funding acquisition, Jeff Niederdeppe – conceptualization; Writing – review & editing, Sarah Gollust – conceptualization; Writing – review & editing; Supervision; Funding acquisition.

## Financial disclosure

Funding for this work was provided by the 10.13039/100000867Robert Wood Johnson Foundation, grant ID 75347.

## Declaration of competing interest

The authors express no conflicting financial or personal relationships that could inappropriately bias this work.

## Footnotes

1 Exploratory analyses reveal statistically significant relationships (p < 0.05) between stories discussing paid leave policy and these stories airing in a location where paid leave policy was introduced or passed. We include these results in tables in supplementary appendix 4. The goal of this study was not to directly assess the relationship between media coverage and policy activity. To do so, we might have chosen a study design focused on specific events (i.e., sampling coverage surrounding the introduction or passage of a law) rather than drawing a representative sample through a constructed week. Further, our study design, with media markets that may span multiple states and differ in the number of stations they include, limits the interpretation of these findings. For example, it could be the case that media markets with more stations, and thus more opportunities for policy-relevant coverage, confound the relationships between news story features and state events. Considering this, we do not present these statistical associations as main findings in this study.
