# Supplementary material for: Paid family leave on local television news in the United States: Setting the agenda for policy reform
Source: SSM Popul Health. 2021 May 15;14:100821. doi: 10.1016/j.ssmph.2021.100821 (PMC8164082; doi:10.1016/j.ssmph.2021.100821)
Supplement: Multimedia component 1 [file mmc1.docx]

**Supplementary appendix 1 | Sampling frame**

Early evening (4-7p) and late evening (10-11:59p) broadcasts

Limited to four major networks available in most markets (ABC, NBC, CBS, and FOX)

M – T – W – Th – F of content per month (ideally, non – consecutive days)

10 months of data collection

**Supplementary appendix 2 | ICR values for variables**

| **Variable** | **Krippendorff’s Alpha** |
| --- | --- |
| *Type of Leave Reference* | |
| Paid leave | .718 |
| Family leave | .931 |
| Medical leave | .859 |
| Sick leave | .974 |
| Parental leave | .908 |
| Paternity leave | .662 |
| Maternity leave | .890 |
| *Policy-Relevance* | |
| Stories referencing public policy | .929 |
| Stories referencing a governmental budget | .720 |
| Details about the policy process included | .896 |
| Policy idea | 1 |
| Draft bill introduced, discussed | .686 |
| Passed a vote | 1 |
| Did not pass a vote | 1 |
| Going for executive approval | 1 |
| Signed into law | .660 |
| *Partisan Sources* | |
| Any of Trump, Republican, or Democrat | .906 |
| President Trump | .911 |
| Democrats | .853 |
| Republicans | .800 |
| Republicans or President Trump | .899 |
| *Other Sources* | |
| Any politician | .839 |
| Government representative, non-elected official | .781 |
| Advocate, lobbyist, interest group | .661 |
| Regular person/community member | .684 |
| Researcher | 1 |
| Doctor, nurse, or other health care professional | 1 |
| Social worker/social service provider | 1 |
| *Exemplars* | |
| Exemplar included | .784 |
| Exemplar visualized | .781 |
| *Location* | |
| Location referenced | .646 |

**Supplementary appendix 3 | Volume of paid leave keyword hits during data collection**

**Supplementary appendix 4 | Exploratory analyses of association between policy-related stories and policy activity in media markets where content aired**

| *4a. Association between policy introduction in dominant state for media market and stories discussing paid leave policy* | | | |
| --- | --- | --- | --- |
| N  Row %  Col % | Not Applicable  [State-level Paid leave policy in place} | Paid leave policy NOT introduced | Paid leave policy introduced |
| Stories discussing paid leave in context of public policy | 37  10.42  60.66 | 31  8.73  35.63 | 287  80.85  70.17 |
| Stories discussing paid leave, not in context of public policy | 24  11.88  39.34 | 56  27.72  64.37 | 122  69.40  29.83 |
| P = 0.000  Pearson chi2 = 37.3072 | | | |

Among stories discussing paid leave in the context of public policy, 287 aired in media markets where paid leave policy was introduced in the dominant state for the media market during the 2018 – 2019 legislative session.

| *4b. Association between policy passage in dominant state for media market and stories discussing paid leave policy* | | | |
| --- | --- | --- | --- |
| N  Row %  Col % | Not Applicable  [State-level Paid leave policy in place} | Paid leave policy NOT passed | Paid leave policy passed |
| Stories discussing paid leave in context of public policy | 37  10.42  60.66 | 210  59.15  60.17 | 108  38.42  73.47 |
| Stories discussing paid leave, not in context of public policy | 24  11.88  39.34 | 139  68.81  39.83 | 39  19.31  26.53 |
| P = 0.017  Pearson chi2 = 8.1937 | | | |

Among stories discussing paid leave in the context of public policy, 108 aired in media markets where paid leave policy was passed in the dominant state for the media market during the 2018 – 2019 legislative session.
